# Pregnancy after bariatric surgery: Consensus recommendations for periconception, antenatal and postnatal care

**DOI:** 10.1111/obr.12927

**Published:** 2019-08-16

**Authors:** Jill Shawe, Dries Ceulemans, Zainab Akhter, Karl Neff, Kathryn Hart, Nicola Heslehurst, Iztok Štotl, Sanjay Agrawal, Regine Steegers‐Theunissen, Shahrad Taheri, Beth Greenslade, Judith Rankin, Bobby Huda, Isy Douek, Sander Galjaard, Orit Blumenfeld, Ann Robinson, Martin Whyte, Elaine Mathews, Roland Devlieger

**Affiliations:** ^1^ Faculty of Health & Human Sciences University of Plymouth Devon UK; ^2^ Department of Development and Regeneration KU Leuven Leuven Belgium; ^3^ Department of Obstetrics and Gynaecology University Hospitals Leuven Leuven Belgium; ^4^ Institute of Health and Society Newcastle University Newcastle upon Tyne UK; ^5^ King's College Hospital NHS Foundation Trust London UK; ^6^ Department of Nutritional Science, Faculty of Health and Medicine University of Surrey Guildford UK; ^7^ Department of Endocrinology, Diabetes and Metabolic Diseases University Medical Centre Ljubljana Slovenia; ^8^ Department of Upper Gastrointestinal and Bariatric Surgery Homerton University Hospital London UK; ^9^ Department of Obstetrics and Gynaecology, Division of Obstetrics and Prenatal Medicine Erasmus MC Rotterdam the Netherlands; ^10^ Weill Cornell Medicine in Qatar Qatar Foundation, Education City Doha Qatar; ^11^ Musgrove Park Hospital Taunton UK; ^12^ Department of Diabetes and Metabolism, St. Bartholomew's Hospital and The Royal London Hospital Barts Health NHS Trust London UK; ^13^ Israel Centre for Disease Control Ministry of Health Jerusalem Israel; ^14^ Faculty of Health and Medical Sciences University of Surrey Guildford UK; ^15^ Department of Clinical and Experimental Medicine University of Surrey Guildford UK; ^16^ St. Richard's Hospital Bariatric Surgery Service, Chichester Western Sussex NHS Foundation Trust Chichester UK; ^17^ Department of Obstetrics, Gynaecology and Reproduction St‐Augustinus Hospital Wilrijk Wilrijk Belgium

**Keywords:** bariatric surgery, metabolic surgery, obesity, pregnancy, obstetrics, gynaecology

## Abstract

The objective of the study is to provide evidence‐based guidance on nutritional management and optimal care for pregnancy after bariatric surgery. A consensus meeting of international and multidisciplinary experts was held to identify relevant research questions in relation to pregnancy after bariatric surgery. A systematic search of available literature was performed, and the ADAPTE protocol for guideline development followed. All available evidence was graded and further discussed during group meetings to formulate recommendations. Where evidence of sufficient quality was lacking, the group made consensus recommendations based on expert clinical experience. The main outcome measures are timing of pregnancy, contraceptive choice, nutritional advice and supplementation, clinical follow‐up of pregnancy, and breastfeeding. We provide recommendations for periconception, antenatal, and postnatal care for women following surgery. These recommendations are summarized in a table and print‐friendly format. Women of reproductive age with a history of bariatric surgery should receive specialized care regarding their reproductive health. Many recommendations are not supported by high‐quality evidence and warrant further research. These areas are highlighted in the paper.

**Abbreviations**
ADA: American Diabetes Association
AGB: Adjustable gastric band
BMI: Body mass index
BPD: Biliopancreatic diversion
BS: Bariatric surgery
CBG: Capillary blood glucose
CGM: Continuous glucose monitoring
COC: Combined oral contraception
FGR: Fetal growth restriction
FPG: Fasting plasma glucose
GDM: Gestational diabetes mellitus
GI: Glycaemic index
GWG: Gestational weight gain
INR: International normalized ratio
IOM: Institute of Medicine
IUD: Intrauterine device
IUS: Intrauterine system
LARC: Long‐acting reversible contraception
LGA: Large for gestational age
NICU: Neonatal intensive care unit
OGTT: Oral glucose tolerance testing
PHH: Postprandial hyperinsulinaemic hypoglycaemia
PTH: Parathyroid hormone
RYGB: Roux‐en‐Y gastric bypass
SG: Sleeve gastrectomy
SGA: Small for gestational age
T2DM: Type 2 diabetes
WHO: World Health Organization

## 1 INTRODUCTION

The prevalence of obesity worldwide has nearly tripled between 1975 and 2016. In 2016, 1.9 billion adults aged 18 years or older (40% of women and 39% of men) were affected by overweight (BMI 25‐29 kg/m^2^) with 650 million (11% men and 15% women) having obesity (BMI ≥ 30 kg/m^2^).^1^ Obesity increases complications for both mother and offspring during pregnancy and childbirth.^2^ Furthermore, there is growing evidence that parental nutrition and lifestyle affect embryonic development with potential long‐term health implications for the infant through on the process of developmental programming.^3^, ^4^ As such, it is generally recommended that both women and men with obesity lose weight before conception.^5^, ^6^ Based on international guidelines,^7^ patients with class III obesity (BMI ≥ 40 kg/m^2^) or class II obesity (BMI 35‐39 kg/m^2^) with associated comorbidities may be eligible for bariatric surgery (BS). Poor success with weight loss by diet alone has led to BS becoming increasingly popular.^8^ Common procedures include (1) sleeve gastrectomy (SG), the most frequently performed operation,^9^ in which the greater curvature of the stomach is resected, reducing stomach volume by 75%, thus limiting food intake. This procedure also removes ghrelin‐producing secreting endocrine cells present in the greater curvature of the stomach, which aid in appetite reduction. Weight loss as well as alterations in other metabolic hormones results in the improvement of glucose homeostasis and results in positive effects on comorbidities therefore reducing appetite and aiding in subsequent diabetes remission.^10^ (2) Roux‐en‐Y gastric bypass (RYGB), a mixed procedure in which the volume of the stomach is reduced to approximately 15 to 30 mL and the absorption of nutrients, is impaired by bypassing part of the small intestine and diverting the food flow to the distant small intestine. This approach not only results in a limited oral intake but also induces malabsorption, although this is reduced over time because of intestinal hypertrophy. Furthermore, an increase in gut hormone secretion (including GLP‐1 and PYY) hormones associated with RYGB may diminish appetite and result in better glucose homeostasis.^11^ (3) Adjustable gastric band (AGB) procedures where an inflatable restrictive band is placed around the upper portion of the stomach creating a small pouch with a narrow opening to the lower stomach, adjusted by adding or removing fluid to the band via a subdermal port. This reduces stomach capacity and appetite.^12^ Other types of surgery include biliopancreatic diversion with duodenal switch, intragastric balloon, and vertical banded gastroplasty, but these are outdated or rarely performed.

As a result of weight loss and enteroendocrine alteration, BS has also been shown to reduce the incidence of obesity‐related comorbidities and complications.^13^ BS is however associated with a potential increase in adverse events due to surgical complications and micronutrient deficiencies and derangements in (neuro)endocrine and metabolic homeostasis.^14^, ^15^, ^16^, ^17^ Approximately 80% of BS is in women, many of whom are of reproductive age.^18^, ^19^, ^20^ BS may improve fertility through restoration of ovulation, and pregnancies after BS are becoming increasingly common.^21^ It has been recognized that changes in gut anatomy and physiology with potential for malnutrition incur increased potential for adverse perinatal outcomes such as small for gestational age (SGA), preterm birth, congenital abnormalities, and perinatal mortality. Pregnancy soon after surgery may increase risk of maternal morbidity and/or mortality.^22^

A need for more specific guidance and nutritional management was recognized, and an international group of experts was assembled to review the available evidence and provide recommendations on the periconception, antenatal, and postnatal care of pregnancies after BS.

## 2 METHODS

An expert meeting focused on pregnancy after BS was organized at the University of Surrey, UK in April 2017 with a follow‐up meeting at University Hospital Leuven, Belgium, in November 2017. These meetings brought together national and international expertise from a multidisciplinary group of researchers and clinicians including specialists in obstetrics and gynaecology, bariatric surgery, endocrinology, dietetics, nutrition, nursing and midwifery, health psychology, epidemiology, and public health. Additional international colleagues were able to join both meetings through teleconferencing.

The objectives of the meetings were to discuss the key questions, to advance scientific knowledge and practice in the area of pregnancy after BS, and to identify key areas of focus for collaborative work to produce consensus clinical guidelines on best practice for facilitating healthy pregnancies after BS.

The clinical guideline was developed using the structure from ADAPTE.^23^ The group formulated specific clinical questions in relation to pregnancy after BS (Table 1). For each question, a systematic search of the available literature was performed, identifying articles published from inception to July 2018. Search terms related to pregnancy (“pregnancy,” “prepregnancy,” “mother,” “maternal,” “conception,” “preconception,” “gravid,” “pregravid”) were combined with terms related to BS (“bariatric surgery,” “weight loss surgery,” “gastric bypass,” “Roux‐en‐Y,” “RYGB,” “sleeve gastrectomy,” “gastric sleeve,” “gastroplasty,” “gastric band,” “LAGB,” “biliopancreatic diversion,” “BPD,” “duodenal switch”) and terms specific for each clinical question. Articles resulting from these searches and relevant references cited in those articles were reviewed. All evidence was graded (Table 2)^24^ and discussed during group meetings. When evidence of sufficient quality was lacking, the group made consensus recommendations based on expert clinical experience. Consensus on the guidelines was declared when 100% of the group agreed with the recommendations. The final document was reviewed by all authors. The recommendations made by this group are summarized in Table 3.

**Table 1:** Clinical questions to be answered in this guideline

Clinical Questions to be Answered in This Guideline
What is the recommended time interval between bariatric surgery and conception?
What types of contraception should be advised to women after bariatric surgery?
Are there special recommendations regarding dietary behaviour?
Which micronutrients should be monitored? Which types of supplements should be prescribed?
Should patients be screened for gestational diabetes and how should they be screened?
Which medical and surgical complications should be monitored, and can they be prevented?
Is breastmilk composition affected by bariatric surgery and can it safely be recommended to patients?

**Table 2:** Type and level of evidence^24^

Quality and Level of Evidence	
1++	High‐quality meta‐analyses, systematic reviews of RCTs, or RCTs (including cluster RCTs) with a very low risk of bias
1+	Well‐conducted meta‐analyses, systematic reviews of RCTs, or RCTs (including cluster RCTs) with a low risk of bias
1–	Meta‐analyses, systematic reviews of RCTs, or RCTs (including cluster RCTs) with a high risk of bias
2++	High‐quality systematic reviews of these types of studies, or individual, non‐RCTs, case‐control studies, cohort studies, CBA studies, ITS, and correlation studies with a very low risk of confounding, bias or chance, and a high probability that the relationship is causal
2+	Well‐conducted non‐RCTs, case‐control studies, cohort studies, CBA studies, ITS, and correlation studies with a low risk of confounding, bias or chance and a moderate probability that the relationship is causal
2–	Non‐RCTs, case‐control studies, cohort studies, CBA studies, ITS and correlation studies with a high risk—or chance—of confounding bias, and a significant risk that the relationship is not causal
3	Non‐analytic studies (for example, case reports, case series)
4	Expert opinion, formal consensus

**Table 3:** Recommendations for clinical practice

Summary of Recommendations	Periconception	First Trimester	Second Trimester	Third Trimester	Postpartum/Breastfeeding
Surgery‐to‐conception interval	Postpone pregnancy until a stable weight is achieved (level 2++)				
Contraception	Counsel women regarding contraception prior to surgery (level 2−) Avoid COCs (level 2+), and encourage the use of LARCs (level 2−)				Counsel women regarding contraception (level 2−) Avoid COCs (level 2+), and encourage the use of LARCs (level 2−)
Nutritional advice	Energy requirements should be individualized on the basis of prepregnancy BMI, GWG, and physical activity level, with limitations on energy dense foods if excessive GWG is identified (level 2−)				
	Provide standard postsurgical dietary advice (level 4)				
	Aim for protein intakes of at least 60 g/day (level 4)				
	Where deranged glucose levels are identified (hyperglycaemia or hypoglycaemia) manipulation of carbohydrate quantity, and/or quality may be warranted (level 4)				
	Hyperglycaemia—reduce rapidly absorbed carbohydrates. Substitute with protein and low GI alternatives (level 4)				
	Early or late dumping—eliminate rapidly absorbed carbohydrates. Substitute with protein and low GI alternatives, six smaller meals. Use liquids 30 min after meals and lay down after eating (level 2−). Avoid caffeinated or alcoholic beverages (level 4) and consider changing eating frequency and portion size (level 4).				
	Artificial nutrition support may be indicated in cases of severe malnutrition during pregnancy, with initiation and choice of feeding route determined by local nutrition support protocols (level 4)				
Nutritional monitoring	Serum indices to be checked every 3 months: full blood count, serum ferritin, and iron studies including transferrin saturation (level 2−), serum folate or red blood cell folate, serum vitamin B12 or transcobalamin (level 2−), serum vitamin A (level 2−). Serum indices to be checked every 6 months: prothrombin time, INR, and serum vitamin K1 concentration (level 2+), serum protein and albumin (level 2−), serum vitamin D with calcium, phosphate, magnesium, and PTH (level 4), renal function and liver function tests (level 4), serum vitamin E (level 4), serum zinc, copper, and selenium (level 4).	Serum indices to be checked every trimester: full blood count, serum ferritin, and iron studies including transferrin saturation (level 2−), serum folate, and serum vitamin B12 (level 2−), serum vitamin A (level 2−), prothrombin time, INR, and serum vitamin K1 concentration (level 2+), serum protein and albumin (level 2−), serum vitamin D with calcium, phosphate, magnesium, and PTH (level 4), renal function and liver function tests (level 4) Extra serum indices to be checked during first trimester: serum vitamin E (level 4), serum zinc, copper, and selenium (level 4).			Serum indices to be checked every 3 months while breastfeeding: full blood count, serum ferritin, and iron studies including transferrin saturation (level 2−), serum folate, and serum vitamin B12 (level 2−), serum vitamin A (level 2−), serum vitamin D with calcium, phosphate, magnesium, and PTH (level 4). Serum indices to be checked every 6 months while breastfeeding: prothrombin time, INR, and serum vitamin K1 concentration (level 2+), serum protein and albumin (level 2−), renal function and liver function tests (level 4), serum vitamin E (level 4), serum zinc, copper, and selenium (level 4).
Nutritional supplementation	Prepregnancy multivitamin and mineral supplement to ensure total daily dosing from all supplements, eg, Table 3 (level 4). Folic acid 0.4 mg daily during preconception and first trimester, 4‐5 mg if obese or diabetic (level 4). Convert Vitamin A to beta‐carotene form (level 2+). Add oral dose of vitamin K weekly if deficiency is noted with coagulation defect (level 2−). Vitamin B12 supplementation (1 mg IM 3 monthly) (level 4). Oral supplementation can be attempted, but reduced absorption is to be expected (level 4). Supplement vitamin D to keep levels above 50 nmol/L, and serum PTH within normal levels (level 4). Add calcium as needed (level 4). Additional supplementation should be given if deficiency is identified.	Thiamine 300 mg daily with two vitamin B compound strong tablets three times daily if vomiting. Prolonged vomiting may require intravenous thiamine and vitamin B complex supplementation (level 3). Give folic acid at a dose of 0.4 mg daily during preconception and first trimester, 4‐5 mg if obese or diabetic (level 4). Further supplementation as during preconception period.			
Diabetes screening	Monitor HbA1c every 3 months in the absence of haemoglobinopathies. If haemoglobin is abnormal then monitor with fasting glucose +/− OGTT. Less frequent testing can be considered if the woman does not have a history of diabetes, according to local policies (level 4).	Check fasting glucose/HbA1c if there is a personal history of diabetes or if other risk factors are present. Treat as T2DM if HbA1c ≥6.5% and/or FPG ≥7.0 mmol/L (level 4).	OGTT at 24‐28 weeks for women who have had AGB (level 4). For all other women either seven‐point CBG profiles or CGM for 1 week between 24 and 28 weeks of gestation (level 4). Repeat HbA1c if there is a personal history of diabetes (level 4).	Repeat screening if clinical suspicion of diabetes (level 4).	Offer screening to patients with GDM. Screen other patients according to local policies or as clinically indicated (level 4).
AGB management		Deflate in case of hyperemesis to prevent band slippage and nutrient deficiencies (level 3).	Assess GWG and fetal growth and manage band as appropriate (level 2++).	Assess GWG and fetal growth and manage band as appropriate (level 2++).	After establishment of lactation, return band to prepregnancy levels (level 2+).
Surgical complications	Excess vomiting—AGB deflation in symptomatic women only to prevent band slippage and/or nutrient requirements not being met (level 3). In case of RYGB, patients should seek medical attention upon onset of abdominal symptoms—timely recognition and early surgical intervention of internal herniation is associated with reduced risk of adverse maternal and fetal outcomes (level 2++).				
Weight management	Postpone pregnancy until a stable weight is achieved (level 2++). Measure preconception weight (level 4).	Measure maternal weight (level 4).	Measure maternal weight and assess for excessive or inadequate GWG. If excessive GWG, assess for complications (level 2+). If ABG, assess GWG and fetal growth and manage band as appropriate (level 2++). If insufficient GWG, monitor fetal growth carefully (level 4)		Pregnancy does not affect long‐term weight loss from BS (level 2+).
Ultrasound scans		Perform routine 12‐week scan (routine) (level 4).	AGB should be deflated if fetal growth is compromised (level 2++). Perform routine 20‐week scan congenital anomaly screening (level 4).	Perform monthly fetal growth monitoring scan(s) from viability (level 2+). Assess for developmental problems such as intracranial bleeding (level 3).	
Mental health	Screen for substance abuse and anxiety or other mental health disorders and offer follow‐up if necessary (level 2+). Advise smoking cessation if necessary (level 2−).				
Breastfeeding					Breastfeeding can be recommended to bariatric patients (level 2++). Monitor maternal micronutrients during lactation (level 3).

Abbreviations: COC, combined oral contraceptive; LARC, long‐acting reversible contraception; BMI, body mass index; GWG, gestational weight gain; GI, glycaemic index; PTH, parathyroid hormone; OGTT, oral glucose tolerance test; AGB, adjustable gastric banding; CBG, capillary blood glucose; CGM, continuous glucose monitoring.

## 3 EVIDENCE AND RECOMMENDATIONS

### 3.1 Bariatric surgery to conception interval

The period after BS is characterized by weight loss which may be rapid after SG and RYGB procedures and slower after AGB, once optimal adjustment has been achieved. During this period postsurgery,^25^, ^26^ women are recommended to postpone pregnancy in order to ensure maximal weight loss, weight stabilization, and to reduce the risk of macronutrient and micronutrient deficiencies and electrolyte imbalances.^5^ Evidence in regard to this recommendation is however scarce. We identified 14 studies reporting on the surgery‐to‐conception interval and pregnancy outcomes, but many studies have limitations in methodology thus preventing comparison.

Parent et al^22^ found that a shorter surgery‐to‐birth interval (less than 2 years) was associated with a higher risk for prematurity, SGA, and neonatal intensive care unit (NICU) admission (level 2++), but data on long‐term outcomes were missing. In contrast, Stentebjerg et al^27^ and Nomura et al^28^ found an increased risk for certain pregnancy complications (iron deficiency, excessive gestational weight gain (GWG), and delivery by caesarean section) if the pregnancy was postponed according to this recommendation (level 2+). Norgaard et al^29^ found no difference in the prevalence of SGA prior to, or after, 18 months (level 2++). Other studies also did not find a difference in gestational outcomes according to surgery to conception interval.^27^, ^30^, ^31^, ^32^, ^33^, ^34^, ^35^, ^36^, ^37^

Based on level 2++ evidence, the members of this group recommend postponing pregnancy until a stable weight is achieved. This is typically achieved 1 year after SG or RYGB procedures and 2 years after AGB.

### 3.2 Contraception

Women recommended to postpone pregnancy during the period of rapid weight loss (1‐2 years) require adequate counselling regarding safe and effective contraception.^38^ As obesity is associated with impaired fertility due to metabolic syndrome and PCOS, patients may not be using contraception presurgery. They should be made aware that fertility increases postoperatively, and contraception usage should be discussed (level 2+).^39^ There is sufficient evidence to show that perioperative contraceptive counselling increases the postoperative use of contraception (level 2+).^40^, ^41^ Contraceptive counselling and contraceptive knowledge by health care providers could however be improved (level 2−),^42^, ^43^ as contraceptive use after BS is often suboptimal, with many women using least reliable methods (level 2+).^39^, ^40^, ^44^, ^45^, ^46^, ^47^, ^48^ This is even more important in patients with a history of infertility, as they have been found to be at increased risk for unprotected intercourse without intent to conceive and have higher early postoperative conception rates.^39^

Both RYGB and, to a lesser extent, SG significantly alter the anatomical structure of the gastrointestinal tract, and theoretically, this gut shortening could affect the absorption of oral contraceptives containing an oestrogen component which undergoes metabolism in the upper gut wall. Absorption of ethinylestradiol from the contraceptive pill may be reduced leading to a decrease in efficacy.^49^ Reliability might also decrease due to postoperative side effects and complications such as vomiting and/or diarrhoea; however, there are few data in women after BS. Limited clinical evidence suggests no substantial decrease in effectiveness of oral contraception among women who underwent a biliopancreatic diversion (BPD), a now uncommon procedure, or AGB.^50^, ^51^ However, evidence from pharmacokinetic studies has shown increased contraceptive failure for progestogen oral contraception among women who underwent a jejunoileal bypass (an older procedure).^52^, ^53^ In general, combined oral contraception (COC) may be less reliable after BS (level 2+).^51^ Additionally, many individuals are still affected by obesity after BS, and this represents a relative contraindication for the use of COC, with both factors increasing the risk of venous thromboembolism.^38^ Alternatives found to be unaffected by BS are parenteral long‐acting reversible contraception (LARC) methods such as the copper intrauterine device (IUD), intrauterine systems (IUS), and progestogen implants. They have been found to be highly effective and acceptable to women (level 2−).^54^, ^55^, ^56^, ^57^ For women choosing nonhormonal barrier methods, both male and female condoms may be suitable; however, the contraceptive diaphragm may be difficult to insert correctly and less reliable as it requires refitting after every 3 kg of weight change.^58^

Consensus from available evidence is that women should receive counselling regarding contraception prior to surgery (level 2−). Combined oral contraception containing oestrogen should be avoided after BS (level 2+). The use of long‐acting reversible contraception such as implants, IUD, or IUS should be encouraged and offered as first line following BS (level 2−).

## 4 NUTRITION AND MICRONUTRIENT MONITORING

### 4.1 Nutritional advice

A large proportion of pregnant women have a poor diet,^59^ independent of BS history. The focus should remain on the regular monitoring of diet quality and nutritional status and on encouraging a general healthy dietary pattern and lifestyle.^3^ At the same time, a healthy diet post‐BS may differ in food group proportions from that of the nonsurgical pregnant population. This is due to a greater emphasis on lean protein sources, followed by fruit and vegetables, and lastly starchy carbohydrates, as the main component of the post BS diet. There is little or no evidence‐based specific dietary (food‐based) advice for pregnancies post BS and few published reports of the dietary intakes of this population.^60^ It therefore seems prudent to combine what we know about an appropriate postsurgical diet with the accepted general dietary advice for pregnancy to provide appropriate guidance.

Energy requirements should be individualized on the basis of prepregnancy BMI, GWG, and physical activity level, with limitations on energy‐dense foods if excessive GWG is identified (level 2+).^60^ Beard et al^61^ recommend a minimum of 60 g of protein/day during pregnancy post‐BS (level 4). However, subsequent antenatal achievement of protein requirements is more difficult following bypass operations.^62^ In the nonpregnant postsurgical patient, intakes of up to 1.5 g/kg ideal body weight/day are proposed (up to a maximum of 2.1 g/kg).^63^ How this translates into pregnancy and in particular how ideal body weight should be defined have not been studied.

Exposure to abnormal glucose levels during pregnancy, similar to that seen in nonsurgical women with GDM, warrants dietary intervention. In the case of hyperglycaemia, it is recommended to reduce rapidly absorbed carbohydrates, substituting them with protein and low glycaemic index (GI) alternatives (level 4).

Parenteral nutrition support may be indicated in cases of severe malnutrition during pregnancy^64^ with initiation and choice of feeding route informed by local nutrition support protocols (level 4). In the absence of dietary advice specific to the postsurgery population, women should be encouraged to adhere to national guidelines regarding diet, taking into consideration changes of anatomy due to BS.

### 4.2 Postprandial syndromes (dumping syndromes)

Postprandial syndrome, or dumping syndrome, is a common effect of bariatric and metabolic surgery. Postprandial syndrome (also termed early dumping syndrome) occurs within 60 minutes of ingestion of food, typically rapidly absorbed carbohydrates, producing symptoms including dizziness, flushing, and palpitations. If early dumping is suspected, rapidly absorbed carbohydrates should be avoided. Additionally, liquids should not be taken 30 minutes before and after eating to encourage a slower gastric transit (level 2−).^65^, ^66^ Caffeinated beverages should be avoided, and patients should be advised to eat slowly and chew well. Individualized advice relating to portion sizes and meal/snack frequency and spacing may be helpful alongside education about the GI of different foods (level 4).^66^ Alcohol consumption can precipitate dumping and is in general contraindicated throughout pregnancy.^67^

Late dumping or postprandial hyperinsulinaemic hypoglycaemia (PHH) is far less common, although the exact prevalence remains unclear due to the lack of clear diagnostic criteria.^68^, ^69^, ^70^ PHH characterized by symptomatic hypoglycaemia that occurs after 60 minutes of eating (typically between 60 and 180 minutes postprandial).^71^ This syndrome should be considered in those who have symptoms of hypoglycaemia (eg, altered mental state, anxiety, sweating, or altered sensorium) that occur in parallel with biochemical evidence of hypoglycaemia, and which then resolve on ingestion of carbohydrate (ie, symptoms agree with Whipple's triad).^71^

In general, management of late dumping/PHH requires more careful dietary manipulation (ie, low GI carbohydrates, small carbohydrate portions, carbohydrates mixed with protein, frequent intake of six small meals) and sometimes referral to an endocrinologist for further investigation and medical management (level 4).^72^ There is no specific approach for PPH described in pregnancy, although important glycaemic excursions potentially could affect fetal growth and well‐being.

### 4.3 Nutritional supplementation and monitoring

Men and women after BS have an increased risk to develop micronutrient deficiencies.^73^ In the formulation of this guidance, it is recognized that there is a lack of evidence on the optimal nutritional monitoring and supplementation strategies in pregnancy after BS. We have therefore used data and guidelines for the nonpregnant postoperative population and supplemented this with pregnancy‐specific data when available. It should be noted that we recommend that pregnancy should be planned and that nutritional supplementation should be optimized preferably 3 to 6 months prior to conception (level 4). A multivitamin and mineral supplement should be taken daily prior to conception and throughout pregnancy (level 4). This supplement should contain the following at a minimum: copper (2 mg), zinc (15 mg), selenium (50 μg), folic acid (5 mg), iron (45‐60 mg or >18 mg after AGB), thiamine (>12 mg), vitamin E (15 mg), and beta‐carotene (vitamin A, 5000 IU) (level 4). The retinol form of vitamin A should be avoided during pregnancy due to teratogenicity risk (level 2+),^74^, ^75^ and supplementation should be adjusted to maintain concentrations within normal limits (level 2−).^76^

Given the risk associated with potential deficiencies in the periconception period, the following indices should be checked at least every 3 months in women planning to become pregnant after BS: serum folate or red blood cell folate (level 2−),^77^ serum vitamin B12 or transcobalamin (level 2−),^62^, ^63^, ^73^, ^78^, ^79^ serum ferritin, iron studies (including transferrin saturation), full blood count (level 2−),^63^, ^73^, ^76^, ^78^, ^79^ and serum vitamin A levels (level 2−).^76^, ^80^, ^81^ In addition, the following should be monitored every 6 months: prothrombin time, international normalized ratio (INR) (level 2+),^82^, ^83^ serum 25‐hydroxyvitamin D with calcium, phosphate, magnesium, and parathyroid hormone (PTH) (level 4), serum protein and albumin (level 2−),^62^, ^78^ renal function and liver function tests (level 4), serum vitamin E (level 4), serum zinc, copper, and selenium (level 4). Serum vitamin K1 concentration should be monitored if coagulation studies are abnormal (level 2+).^83^

Specific supplementation is recommended in the preconception and periconception period (Tables 3 and 4). In most patients after BS, 0.4 mg per day of folic acid is sufficient as doses >0.3 mg are not absorbed, due to lack of dihydrofolate reductase in intestinal cells. Despite having undergone BS, many patients still have a BMI > 30 kg/m^2^. Current guidelines suggest that additional folic acid at a dose of 4 or 5 mg daily should be given to these patients during the periconception period and throughout the first trimester (level 4).^84^ Postsurgery vitamin B12 regimens should be continued preconception at a dose of 1 mg every 3 months via intramuscular depot injection. Alternatively, oral supplementation (1 mg/day) can be used to increase compliance in the patient. However, a reduced absorption is to be expected as the secretion of intrinsic factor is diminished (level 4).^85^ Additional vitamin B12 supplementation should be given as needed to maintain serum concentrations within normal limits (level 4). Iron supplementation should be continued at a minimum dose of 45 mg of elemental iron daily (>18 mg for AGB); this should be increased as needed to maintain ferritin within normal limits (level 4). Vitamin D should be supplemented to maintain a concentration of 50 nmol/L or greater with a serum PTH within normal limits (level 4). Calcium should be added to on‐going vitamin D supplementation as needed to maintain PTH within normal limits (level 4). If vitamin K1 deficiency is measured or suggested by coagulation defects, it is advised to supplement this with an oral dose of 10 mg weekly (level 2+).^83^

**Table 4:** Daily dose recommendations for (pre)pregnancy supplementation

Daily Dose Recommendations for (Pre)pregnancy Supplementation (Level 4)
Thiamine >12 mg
Folic acid 0.4 mg daily, during preconception and first trimester, 4‐5 mg if obese or diabetic
Calcium 1200‐1500 mg in divided doses (includes dietary intake)
Vitamin D >40 mcg (1000 IU)
Iron 45‐60 mg elemental iron (AGB >18 mg)
Copper 2 mg (AGB >1 mg)
Zinc 8‐15 mg per 1 mg copper
Vitamin K 90‐120 μg
Vitamin E 15 mg
Vitamin A 5000 IU, should be in B carotene form in pregnancy
Selenium 50 μg daily

Abbreviations: IU, international units; AGB, adjustable gastric banding.

During pregnancy, serum levels of many micronutrients and macronutrients will decrease as a result of the expanding maternal blood volume and increasing demands of the growing fetus. Therefore, it is recommended to check the following indices at least once per trimester and use pregnancy‐specific ranges: serum folate (level 2−)^77^; serum vitamin B12 (level 2−)^62^, ^63^, ^73^, ^78^, ^79^; serum ferritin, iron studies including transferrin saturation and full blood count (level 2−)^63^, ^73^, ^76^, ^78^, ^79^; serum vitamin D with calcium, phosphate, magnesium, and PTH (level 4); serum vitamin A (level 2−)^76^, ^80^, ^81^; prothrombin time, INR, and serum vitamin K1 concentration (level 2+)^82^, ^83^; serum protein and albumin (level 2−)^62^, ^78^; and renal function and liver function tests (level 4). In addition, we advise to monitor serum vitamin E, serum zinc, copper, and selenium (level 4) during the first trimester.

During pregnancy, thiamine 300 mg daily with vitamin B complex should be prescribed if prolonged vomiting occurs due to hyperemesis or other causes (level 3).^86^, ^87^, ^88^

Furthermore, intravenous thiamine should be given at a minimum dose of 100 mg daily with intravenous vitamin B complex if oral supplementation is not possible due to the severity of vomiting (level 3).^86^, ^87^, ^88^ Further supplementation in regards to vitamin B12, iron, vitamin D, calcium, vitamin A, and vitamin K should be provided as in the preconception period (level 4).

Our recommendations for preconception nutritional supplementation generally agree with the British Obesity and Metabolic Surgery Society (BOMSS) and the American Society of Metabolic and Bariatric Surgeons (ASMBS) recommendations^63^, ^84^ and represent the commonly agreed standard of care with regards to micronutrient replacement.

### 4.4 Breastfeeding

Limited data are available on breastfeeding after BS. In longitudinal studies, the composition of breastmilk from women after BS was found to be largely comparable with women without prior BS (level 2++).^89^, ^90^ Gimenes et al^91^ found children born to mothers who had undergone BS and who were breastfed for at least 6 months to have lower fat mass and lower glucose levels, possibly protecting them from the development of obesity later in life. These authors therefore recommend breastfeeding in these women for at least 6 months in accordance to the general WHO guidelines (level 2+).^92^ Case reports have demonstrated adverse maternal and/or neonatal outcomes due to micronutrient deficiencies during lactation (level 3).^93^, ^94^, ^95^ Therefore, we advise supporting women wishing to breastfeed after BS (level 2+) and suggest that their nutritional status is closely monitored during lactation with additional supplements to those routinely advised after BS prescribed when necessary (level 3).

## 5 ASSESSMENT AND PREVENTION OF MEDICAL COMPLICATIONS

### 5.1 Ultrasound monitoring of fetal growth and anomalies

Most types of BS have been found to double the risk of fetal growth restriction (FGR) and SGA infants in comparison with BMI‐matched women^96^ and women with obesity.^97^ This risk is higher with procedures that potentially further induce malabsorption (such as RYGB), when compared with procedures such as AGB or SG (level 2+).^96^, ^98^ Studies suggest that it would seem preferable for women of reproductive age to consider more restrictive procedures to limit this risk. AGB is however also associated with lower birth weight when the band remains inflated during pregnancy (level 2++).^99^ Ultrasound monitoring of fetal growth should be offered to all women with a history of BS (level 2++). We recommend monthly screening from viability, especially in the presence of additional risk factors (eg, smokers, low GWG, teenagers) (level 4).

It is still unclear whether BS increases the risk for congenital malformations in the offspring as strong epidemiological data are lacking.^33^ Several case reports and case studies have reported on the association between nutritional deficiencies in the mother and congenital anomalies in the offspring (level 3).^83^, ^100^, ^101^, ^102^, ^103^, ^104^ We therefore suggest an additional detailed anomaly scan during the late first or second trimester, especially in women with nutritional deficiencies (level 3), and sonographic follow‐up of fetal growth during the third trimester (level 2++).

### 5.2 Weight management in pregnancy

Weight regain following BS is a known problem in a substantial number of patients.^105^, ^106^, ^107^ It is therefore important to avoid excessive GWG and postpartum weight retention in women after BS. On the other hand, insufficient GWG increases the risk for FGR and low birth weight.^108^ So far, no specific guidelines for GWG during pregnancy in postbariatric women are available and few studies have focussed on the subject.

Overall, women with a history of BS gain less weight during pregnancy compared with women without prior BS, especially during the third trimester (level 2++).^27^, ^109^, ^110^, ^111^, ^112^, ^113^ Women who conceive within 18 months after surgery also appear to have less GWG in comparison with those who conceive after this period (level 2+).^27^ Sheiner et al^114^ compared GWG between different types of surgery and found a reduced GWG for vertical banded gastroplasty and silastic ring vertical gastroplasty when compared with RYGB, and higher GWG for AGB compared with all other forms of BS (level 2+).

Studies correlating GWG and pregnancy outcome are scarce. Ducarme et al^111^ reported a significant reduction in both low birth weight (<10% centile) and macrosomia (>90% centile) after AGB compared with controls with obesity, despite lower mean GWG. In a small retrospective cohort, Santulli et al^115^ reported no clear relation between birth weight and GWG in women after RYGB (level 2−). Stentebjerg et al^27^ explored differences in outcome between women who gained appropriate, inadequate, or excessive weight according to the Institute of Medicine (IOM) guidelines for pregnant women.^116^ GWG exceeding the guidelines increased the risk for preeclampsia and low Apgar scores at 1 minute (level 2+). Women with GWG below the guidelines delivered the smallest children. Lapolla et al^112^ found a similar trend towards smaller children if GWG was below the guidelines. As pregnancy does not appear to affect long‐term weight in women with a history of BS (level 2+)^25^, ^26^ and in view of the strong correlation between insufficient GWG, adverse neonatal outcomes, and increased risk of low birth weight in the general population, we advise women with a history of BS to adhere to the IOM guidelines (level 2+).

In women with AGB, evidence regarding band management and weight gain during pregnancy is also limited. Active band management appears to facilitate adherence to the IOM guidelines and was not associated with low birth weight (level 2++).^26^, ^99^, ^109^, ^117^ In contrast, band deflation was associated with macrosomia (level 3).^117^

We recommend health professionals caring for women after BS to measure BMI and monitor GWG in order to advise regarding adequate GWG relating to their prepregnancy BMI in accordance to the IOM guidelines (level 2+). If GWG is excessive, women should be assessed for complications (level 2+). In the case of insufficient GWG, diet should be revised and fetal growth carefully monitored (level 4).

### 5.3 Diabetes screening

Currently, there are no specific guidelines on screening and treatment for diabetes during pregnancy in women after BS. The risk of developing type 2 diabetes (T2DM) and GDM is reduced in women after BS when compared with women without BS matched for their preoperative BMI.^118^ In contrast, women who have undergone BS are often still affected by obesity or overweight and remain at higher risk for T2DM and GDM than women with a healthy weight without BS.^96^, ^119^ Undiagnosed diabetes in pregnancy results in an increased risk for adverse outcomes including fetal anomalies.^120^

Women who are planning to become pregnant post‐BS should be screened for preexisting diabetes in the prepregnancy period, so that it can be identified and treated prior to conception (level 4). During pregnancy, women with a history of BS should routinely be screened for GDM (level 4).^121^, ^122^ Patients with other risk factors for developing GDM should be offered early screening according to local policies to exclude preexisting diabetes. This is best performed using fasting plasma glucose (FPG) or Hba1c (level 4). As data on cut‐off values during pregnancies after bariatric surgery are lacking, we recommended using the guideline from the American Diabetes Association (ADA).^123^ As such, the diagnosis of T2DM is made if HbA1c and/or FPG is greater than or equal to 6.5% and greater than or equal to 7.0 mmol/L, respectively. Care should be taken when using HbA1c as it is less sensitive to screen for T2DM and GDM using this method when compared with FPG. In addition, the HAPO study showed that associations with adverse outcomes were significantly stronger with glucose measures than with Hba1c.^124^ However, this is offset by the test's greater practicality as it can be used in the nonfasting state, and the wider application of a more convenient test may increase the number of diagnoses made.

Oral glucose tolerance testing (OGTT) is appropriate for women with AGB and can be used to screen for GDM between 24 and 28 weeks (level 4).^122^ However, given the physiological changes associated with RYGB, SG, and BPD, there are valid concerns with regards to the tolerability (dumping) and accuracy of OGTT in these women (level 2−).^121^, ^122^, ^125^ Studies have suggested that using either a seven‐point capillary blood glucose (CBG) profile or continuous glucose monitoring (CGM) or for 1 week between 24 and 28 weeks is the most appropriate method for GDM screening in these women (level 4). However, appropriate threshold values for random capillary glucose thresholds need yet to be defined in the post‐BS population.

In the absence of specific outcome data for the post‐BS population, it seems reasonable to aim for the same targets as used in the general population with GDM according to NICE^126^ or according to local policies, that is maintaining capillary blood glucose concentrations below 5.3 mmol/L fasting, 7.8 mmol/L 1 hour after eating, and 6.4 mmol/L 2 hours after eating, if these goals can be achieved without hypoglycaemia (level 4). In women with a history of T2DM that is in remission postoperatively, additional value may be gained from screening with fasting glucose or HbA1c at booking and in the second trimester (level 4).^121^ Screening in the third trimester should also be considered if there is a clinical suspicion of the interval development of diabetes (such as accelerated fetal growth indices).

If the diagnosis of GDM is made, it should be treated according to local policies (level 4). In general, this consists of lifestyle interventions first. If glycaemic targets are not met after 1 to 2 weeks, pharmacological treatment should be considered.^127^

### 5.4 Mental health

BS is associated with an increased risk for mental health problems and substance abuse.^128^, ^129^, ^130^ Data on mental health and substance abuse during pregnancies after BS are very limited. Higher anxiety rates during pregnancy are reported, without significant increase in depression rates (level 2+).^131^ We found no data on postpartum depression following BS. Guelinckx et al^60^ reported on maternal smoking during the first trimester of pregnancy in post‐BS women. Overall smoking rate was 24%, without a clear relation to the type of procedure. Smoking prevalence was comparable with the general nonpregnant female population, but much higher than in the general pregnant population in the same region (6%). No studies were found reporting on alcohol or other substance abuse during pregnancies after BS. As such, we recommend health providers to screen for anxiety and other mental health disorders prior and during pregnancy, and follow‐up should be offered when necessary (level 2+). Smoking cessation and alcohol use should be discussed when necessary as per general prepregnancy guidance (level 2−).

### 5.5 Assessment and prevention of surgical complications

Evidence for two common surgical complications during pregnancy was found: internal herniation following RYGB and gastric band slippage following AGB. With regards to internal herniation, an incidence of 8% has been reported during pregnancies after RYGB.^132^ Upper abdominal pain complicates 46% of such pregnancies, and internal herniation is diagnosed in 32.8% of these cases (level 3).^133^ Women reporting abdominal pain had an increased risk of preterm birth and significantly lower birth weight compared with women without abdominal pain, suggesting that severe abdominal pain and abdominal surgery may induce uterine contractions (level 3).^133^ Repeat internal herniation can occur in the same pregnancy even after previous closure of mesenteric defects (level 3). In a review of 22 cases of internal herniation during pregnancy after BS, all patients presented with abdominal pain and half of patients presented with nausea and/or vomiting. The most common location of the hernia was Petersen's space (45.5%), and there was a high incidence of maternal and fetal death in this case series (9% and 13.6%, respectively) (level 2−).^134^ A systematic review reported that all maternal and perinatal deaths in pregnancies complicated by internal herniation after RYGB occurred in women treated later than 48 hours after symptom onset (level 2++).^135^

We recommend that all women with RYGB should be advised about the risks and symptoms of internal herniation and should seek appropriate medical assistance without delay. Care providers should be advised that any pregnant women with a history of RYGB that presents with abdominal pain should be assumed to have a small bowel obstruction due to internal herniation until proven otherwise (level 4)^136^ and that imaging techniques and operative intervention, often performed with reluctance in pregnant women, should not be delayed (level 2++).

Gastric band slippage may be increased during pregnancy due to vomiting and increased intraabdominal pressure. One study reported an incidence of 12% during pregnancy compared with 3% to 5% in the general AGB population (level 3).^137^ A shorter time interval between AGB and pregnancy was associated with a higher rate of primary band revisions after pregnancy (level 2+).^138^ Patients should be counselled on the risk and symptoms of band slip during pregnancy and in the postpartum period (level 4).

### 5.6 Research gaps

The recommendations issued in this review are based on a systematic research of the literature by a multidisciplinary group of international experts. The group has identified areas for which the level of evidence and therefore the quality of the recommendations is largely based on expert opinion. It is felt by the group that following areas need further robust investigation with regard to women and children's health in pregnancy following BS:

- Contraceptive counselling, safety, efficacy, and use
- Timing of pregnancy
- Gestational weight gain recommendations
- Nutrition during pregnancy
- Optimal macronutrient monitoring and substitution/supplementation such as protein intake, including management of supplementation and when parental nutrition should be considered
- Optimal micronutrient monitoring and substitution
- Prevention and treatment of dumping and PPH
- Monitoring of fetal growth
- Screening and treatment for GDM
- Screening and treatment of surgical complications
- Mental health and substance abuse

## 6 CONCLUSIONS

**Figure 1:**
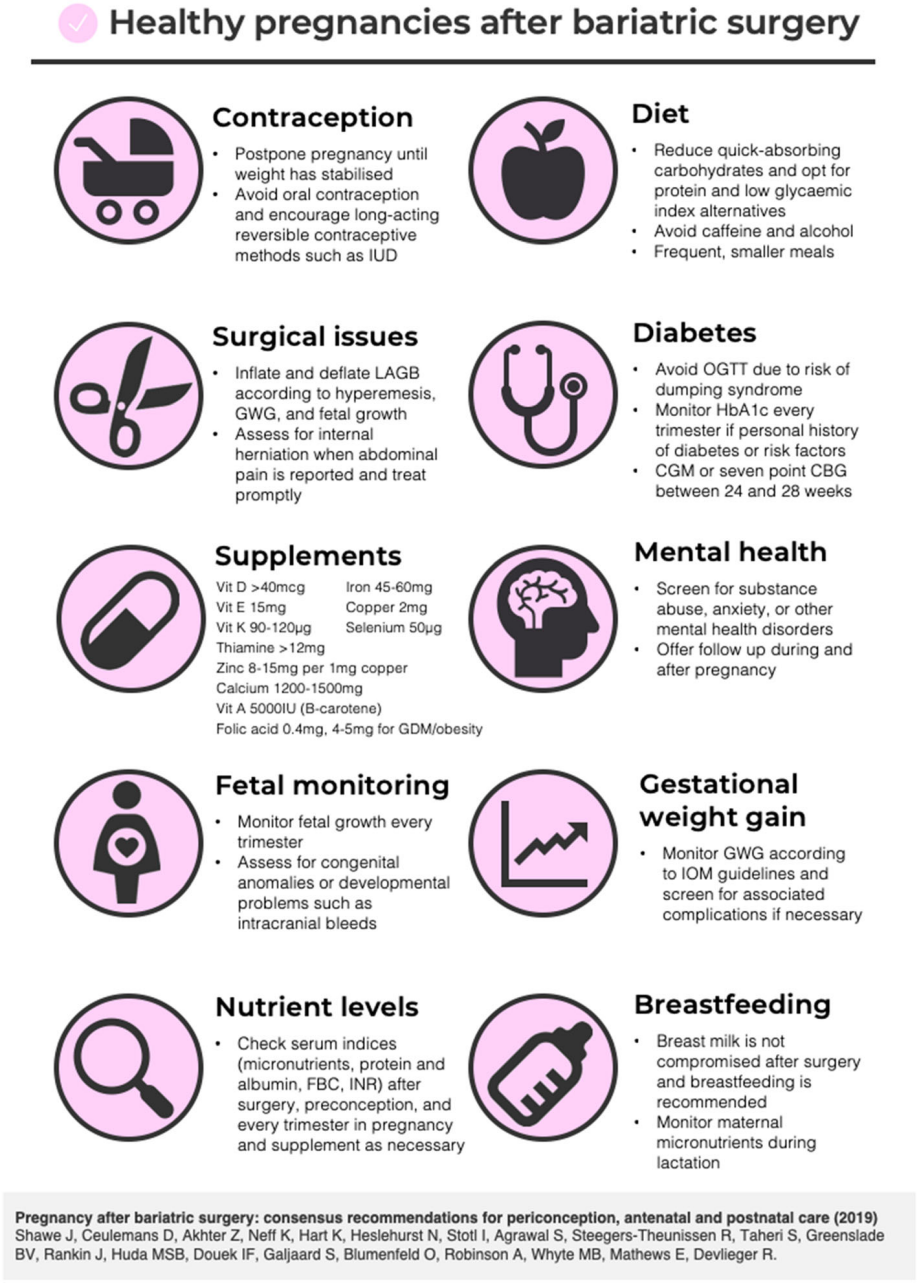
Print‐friendly presentation of the recommendations for healthy pregnancies after bariatric surgery. [Colour figure can be viewed at http://wileyonlinelibrary.com]

This review summarizes current recommendations on the periconception, antenatal, and postnatal care of women following BS. Recommendations on the care of these patients are summarized in Table 3 and presented in a print‐friendly format for practical use in the clinical setting (Figure 1). Our work highlights the paucity of studies on the optimal care for this growing group of women and identifies research gaps in this field. The publication of these guidelines will be the first step in a research collaboration which will address these unanswered questions.

## CONFLICT OF INTEREST

The Institute of Advanced Studies (IAS) provided financial support of the international workshop organized at the University of Surrey, UK. RD received a fundamental clinical investigatorship from FWO Flanders (1803311N). The authors declare no conflict of interest.

## References

[1] WHO . Obesity and overweight. 2018; http://www.who.int/mediacentre/factsheets/fs311/en/. Accessed 11/04/2018, 2018.

[2] Marchi J , Berg M , Dencker A , Olander EK , Begley C . Risks associated with obesity in pregnancy, for the mother and baby: a systematic review of reviews. Obes Rev. 2015;16(8):621‐638. 26016557 10.1111/obr.12288

[3] Barker M , Dombrowski SU , Colbourn T , et al. Intervention strategies to improve nutrition and health behaviours before conception. The Lancet. 2018;391(10132):1853‐1864. 10.1016/S0140-6736(18)30313-1 PMC6075694 29673875

[4] Fleming TP , Watkins AJ , Velazquez MA , et al. Origins of lifetime health around the time of conception: causes and consequences. The Lancet. 2018;391(10132):1842‐1852. 10.1016/S0140-6736(18)30312-X PMC5975952 29673874

[5] ACOG . Committee Opinion No. 549: obesity in pregnancy. Obstet Gynecol. 2013;121(1):213‐217. 23262963 10.1097/01.aog.0000425667.10377.60

[6] Stephenson J , Heslehurst N , Hall J , et al. Before the beginning: nutrition and lifestyle in the preconception period and its importance for future health. The Lancet. 2018;391(10132):1830‐1841. 10.1016/S0140-6736(18)30311-8 PMC6075697 29673873

[7] National Institute for Health and Care Excellence . Obesity: identification, assessment and management. NICE guideline [CG189]. 2014; https://www.nice.org.uk/guidance/cg189. 36719951

[8] Sjöström L , Narbro K , Sjöström CD , et al. Effects of bariatric surgery on mortality in Swedish obese subjects. N Engl J Med. 2007;357(8):741‐752. 17715408 10.1056/NEJMoa066254

[9] Angrisani L , Santonicola A , Iovino P , et al. Bariatric surgery and endoluminal procedures: IFSO Worldwide Survey 2014. Obes Surg. 2017;27(9):2279‐2289. 28405878 10.1007/s11695-017-2666-x PMC5562777

[10] Peterli R , Steinert RE , Woelnerhanssen B , et al. Metabolic and hormonal changes after laparoscopic Roux‐en‐Y gastric bypass and sleeve gastrectomy: a randomized, prospective trial. Obes Surg. 2012;22(5):740‐748. 22354457 10.1007/s11695-012-0622-3 PMC3319900

[11] Andrew CA , Umashanker D , Aronne LJ , Shukla AP . Intestinal and gastric origins for diabetes resolution after bariatric surgery. Curr Obes Rep. 2018;7(2):139‐146. 29637413 10.1007/s13679-018-0302-2

[12] Cobourn CS , Dixon JB . LAGB: The Technique In: Agrawal S, ed. Obesity, Bariatric and Metabolic Surgery. Switzerland: Springer International Publishing; 2016.

[13] Sjostrom L . Review of the key results from the Swedish Obese Subjects (SOS) trial ‐ a prospective controlled intervention study of bariatric surgery. J Intern Med. 2013;273(3):219‐234. 23163728 10.1111/joim.12012

[14] Ikramuddin S , Korner J , Lee WJ , et al. Roux‐en‐Y gastric bypass vs intensive medical management for the control of type 2 diabetes, hypertension, and hyperlipidemia: the Diabetes Surgery Study randomized clinical trial. JAMA. 2013;309(21):2240‐2249. 23736733 10.1001/jama.2013.5835 PMC3954742

[15] Jakobsen GS , Smastuen MC , Sandbu R , et al. Association of Bariatric Surgery vs Medical Obesity Treatment With Long‐term Medical Complications and Obesity‐Related Comorbidities. JAMA. 2018;319(3):291‐301. 29340680 10.1001/jama.2017.21055 PMC5833560

[16] Gloy VL , Briel M , Bhatt DL , et al. Bariatric surgery versus non‐surgical treatment for obesity: a systematic review and meta‐analysis of randomised controlled trials. BMJ. 2013;347(oct22 1):f5934. 24149519 10.1136/bmj.f5934 PMC3806364

[17] Schauer PR , Bhatt DL , Kirwan JP , et al. Bariatric Surgery versus Intensive Medical Therapy for Diabetes — 5‐Year Outcomes. N Engl J Med. 2017;376(7):641‐651. 28199805 10.1056/NEJMoa1600869 PMC5451258

[18] Kizy S , Jahansouz C , Downey MC , Hevelone N , Ikramuddin S , Leslie D . National trends in bariatric surgery 2012–2015: demographics, procedure selection, readmissions, and cost. Obes Surg. 2017;27(11):2933‐2939. 28534189 10.1007/s11695-017-2719-1

[19] Abraham A , Ikramuddin S , Jahansouz C , Arafat F , Hevelone N , Leslie D . Trends in bariatric surgery: procedure selection, revisional surgeries, and readmissions. Obes Surg. 2016;26(7):1371‐1377. 26715330 10.1007/s11695-015-1974-2

[20] Santry HP , Gillen DL , Lauderdale DS . Trends in bariatric surgical procedures. JAMA. 2005;294(15):1909‐1917. 16234497 10.1001/jama.294.15.1909

[21] Maggard MA , Yermilov I , Li Z , et al. Pregnancy and fertility following bariatric surgery: a systematic review. JAMA. 2008;300(19):2286‐2296. 19017915 10.1001/jama.2008.641

[22] Parent B , Martopullo I , Weiss NS , Khandelwal S , Fay EE , Rowhani‐Rahbar A . Bariatric surgery in women of childbearing age, timing between an operation and birth, and associated perinatal complications. JAMA Surg. 2017;152(2):1‐8. 10.1001/jamasurg.2016.3621 27760265

[23] Fervers B , Burgers JS , Voellinger R , et al. Guideline adaptation: an approach to enhance efficiency in guideline development and improve utilisation. BMJ Qual Saf. 2011;20(3):228‐236. 10.1136/bmjqs.2010.043257 21209134

[24] RCOG . Development of RCOG Green‐top Guidelines (Clinical Governance Advice No. 1). 2015.

[25] Alatishe A , Ammori BJ , New JP , Syed AA . Bariatric surgery in women of childbearing age. QJM. 2013;106(8):717‐720. 23576775 10.1093/qjmed/hct081

[26] Quyen Pham T , Pigeyre M , Caiazzo R , Verkindt H , Deruelle P , Pattou F . Does pregnancy influence long‐term results of bariatric surgery? Surg Obes Relat Dis: official journal of the American Society for Bariatric Surgery. 2015;11(5):1134‐1139. 10.1016/j.soard.2015.03.015 26645490

[27] Stentebjerg LL , Andersen LLT , Renault K , Stoving RK , Jensen DM . Pregnancy and perinatal outcomes according to surgery to conception interval and gestational weight gain in women with previous gastric bypass. J Matern Fetal Neonatal Med. 2017;30(10):1182‐1188. 27426696 10.1080/14767058.2016.1208746

[28] Nomura RM , Dias MC , Igai AM , Paiva LV , Zugaib M . Anemia during pregnancy after silastic ring Roux‐en‐Y gastric bypass: influence of time to conception. Obes Surg. 2011;21(4):479‐484. 21336558 10.1007/s11695-011-0376-3

[29] Norgaard LN , Gjerris AC , Kirkegaard I , Berlac JF , Tabor A . Fetal growth in pregnancies conceived after gastric bypass surgery in relation to surgery‐to‐conception interval: a Danish national cohort study. PLoS ONE. 2014;9(3):e90317 10.1371/journal.pone.0090317 24658186 PMC3962335

[30] Wax JR , Cartin A , Wolff R , Lepich S , Pinette MG , Blackstone J . Pregnancy following gastric bypass for morbid obesity: effect of surgery‐to‐conception interval on maternal and neonatal outcomes. Obes Surg. 2008;18(12):1517‐1521. 18685903 10.1007/s11695-008-9647-z

[31] Sheiner E , Edri A , Balaban E , Levi I , Aricha‐Tamir B . Pregnancy outcome of patients who conceive during or after the first year following bariatric surgery. Am J Obstet Gynecol. 2011;204(1):50 e51‐50 e56. 20887972 10.1016/j.ajog.2010.08.027

[32] Ducarme G , Parisio L , Santulli P , Carbillon L , Mandelbrot L , Luton D . Neonatal outcomes in pregnancies after bariatric surgery: a retrospective multi‐centric cohort study in three French referral centers. J Matern Fetal Neonatal Med. 2013;26(3):275‐278. 23043220 10.3109/14767058.2012.735723

[33] Kjaer MM , Nilas L . Pregnancy after bariatric surgery—a review of benefits and risks. Acta Obstet Gynecol Scand. 2013;92(3):264‐271. 23066836 10.1111/aogs.12035

[34] Ducarme G , Chesnoy V , Lemarie P , Koumare S , Krawczykowski D . Pregnancy outcomes after laparoscopic sleeve gastrectomy among obese patients. Int J Gynaecol Obstet. 2015;130(2):127‐131. 25935476 10.1016/j.ijgo.2015.03.022

[35] Chaichian S , Moazzami B , Jesmi F , et al. The controversy of the most proper time for pregnancy after bariatric surgery: a review of ten cases. Obes Surg. 2016;26(6):1352‐1356. 26951153 10.1007/s11695-016-2124-1

[36] Hazart J , Le Guennec D , Accoceberry M , et al. Maternal nutritional deficiencies and small‐for‐gestational‐age neonates at birth of women who have undergone bariatric surgery. J Pregnancy. 2017;2017:1‐11. 10.1155/2017/4168541 PMC5610850 29082043

[37] Yau PO , Parikh M , Saunders JK , Chui P , Zablocki T , Welcome AU . Pregnancy after bariatric surgery: the effect of time‐to‐conception on pregnancy outcomes. Surg Obes Relat Dis: official journal of the American Society for Bariatric Surgery. 2017;13(11):1899‐1905. 10.1016/j.soard.2017.07.015 28797671

[38] The Faculty of Sexual & Reproductive Healthcare . UK Medical Eligibility Criteria for Contraceptive Use (UKMEC). In: 2017.

[39] Menke MN , King WC , White GE , et al. Contraception and conception after bariatric surgery. Obstet Gynecol. 2017;130(5):979‐987. 29016506 10.1097/AOG.0000000000002323 PMC5679259

[40] Casas RS , Tong I , Bourjeily G . Contraceptive use in women having bariatric surgery. J Gen Intern Med. 2014;29:S58.

[41] Mengesha B , Griffin L , Nagle A , Kiley J . Assessment of contraceptive needs in women undergoing bariatric surgery. Contraception. 2016;94(1):74‐77. 26939526 10.1016/j.contraception.2016.02.027

[42] Graham YNH , Mansour D , Small PK , et al. A survey of bariatric surgical and reproductive health professionals' knowledge and provision of contraception to reproductive‐aged bariatric surgical patients. Obes Surg. 2016;26(8):1918‐1923. 26801788 10.1007/s11695-015-2037-4

[43] Jatlaoui TC , Cordes S , Goedken P , Jamieson DJ , Cwiak C . Family planning knowledge, attitudes and practices among bariatric healthcare providers. Contraception. 2016;93(5):455‐462. 26768856 10.1016/j.contraception.2015.12.016

[44] Krishnan S , Hacker M , Haider S , et al. Contraceptive counseling and utilization in women who underwent bariatric surgery. Contraception. 2009;80(2):212.

[45] Gosman GG , King WC , Schrope B , et al. Reproductive health of women electing bariatric surgery. Fertil Steril. 2010;94(4):1426‐1431. 19815190 10.1016/j.fertnstert.2009.08.028 PMC2888936

[46] Mody SK , Hacker MR , Dodge LE , Thornton K , Schneider B , Haider S . Contraceptive counseling for women who undergo bariatric surgery. J Womens Health (Larchmt). 2011;20(12):1785‐1788. 21988600 10.1089/jwh.2010.2704

[47] Ginstman C , Frisk J , Ottosson J , Brynhildsen J . Contraceptive use before and after gastric bypass: a questionnaire study. Obes Surg. 2015;25(11):2066‐2070. 25744304 10.1007/s11695-015-1641-7 PMC4595520

[48] Dabi Y , Thubert T , Benachi A , Ferretti S , Tranchart H , Dagher I . Pregnancies within the first year following sleeve gastrectomy: impact on maternal and fetal outcomes. Eur J Obstet Gynecol Reprod Biol. 2017;212:190‐192. 28238474 10.1016/j.ejogrb.2017.02.009

[49] Schlatter J . Oral contraceptives after bariatric surgery. Obes Facts. 2017;10(2):118‐126. 28433989 10.1159/000449508 PMC5644910

[50] Weiss HG , Nehoda H , Labeck B , Hourmont K , Marth C , Aigner F . Pregnancies after adjustable gastric banding. Obes Surg. 2001;11(3):303‐306. 11433905 10.1381/096089201321336647

[51] Gerrits EG , Ceulemans R , van Hee R , Hendrickx L , Totte E . Contraceptive treatment after biliopancreatic diversion needs consensus. Obes Surg. 2003;13(3):378‐382. 12841897 10.1381/096089203765887697

[52] Victor A , Odlind V , Kral JG . Oral contraceptive absorption and sex hormone binding globulins in obese women: effects of jejunoileal bypass. Gastroenterol Clin North Am. 1987;16(3):483‐491. 2449395

[53] Andersen AN , Lebech PE , Sorensen TI , Borggaard B . Sex hormone levels and intestinal absorption of estradiol and D‐norgestrel in women following bypass surgery for morbid obesity. Int J Obes (Lond). 1982;6(1):91‐96. 7068318

[54] Hillman JB , Miller RJ , Inge TH . Menstrual concerns and intrauterine contraception among adolescent bariatric surgery patients. J Womens Health (Larchmt). 2011;20(4):533‐538. 21413894 10.1089/jwh.2010.2462 PMC3075047

[55] Kanj RV , Schwartz BI , Alexander M , et al. Continuation rates and satisfaction with the levonorgestrel intrauterine device in nulliparous adolescents undergoing bariatric surgery. J Pediatr Adolesc Gynecol. 2016;29(2):203‐204.

[56] Luyssen J , Jans G , Bogaerts A , et al. Contraception, menstruation, and sexuality after bariatric surgery: a prospective cohort study. Obes Surg. 2018;28(5):1385‐1393. 29197048 10.1007/s11695-017-3033-7

[57] Ciangura C , Corigliano N , Basdevant A , et al. Etonorgestrel concentrations in morbidly obese women following Roux‐en‐Y gastric bypass surgery: three case reports. Contraception. 2011;84(6):649‐651. 22078197 10.1016/j.contraception.2011.03.015

[58] Graham Y , Wilkes S , Mansour D , Small PK . Contraceptive needs of women following bariatric surgery. J Fam Plann Reprod Health Care. 2014;40(4):241‐244. 25240027 10.1136/jfprhc-2014-100959

[59] Ma RCW , Schmidt MI , Tam WH , McIntyre HD , Catalano PM . Clinical management of pregnancy in the obese mother: before conception, during pregnancy, and post partum. 2016. 10.1016/S2213-8587(16)30278-9 PMC6691730 27743977

[60] Guelinckx I , Devlieger R , Donceel P , et al. Lifestyle after bariatric surgery: a multicenter, prospective cohort study in pregnant women. Obes Surg. 2012;22(9):1456‐1464. 22644802 10.1007/s11695-012-0675-3

[61] Beard JH , Bell RL , Duffy AJ . Reproductive considerations and pregnancy after bariatric surgery: current evidence and recommendations. Obes Surg. 2008;18(8):1023‐1027. 18392904 10.1007/s11695-007-9389-3

[62] Mead NC , Sakkatos P , Sakellaropoulos GC , Adonakis GL , Alexandrides TK , Kalfarentzos F . Pregnancy outcomes and nutritional indices after 3 types of bariatric surgery performed at a single institution. Surg Obes Relat Dis: official journal of the American Society for Bariatric Surgery. 2014;10(6):1166‐1173. 10.1016/j.soard.2014.02.011 24913588

[63] Mechanick JI , Youdim A , Jones DB , et al. Clinical practice guidelines for the perioperative nutritional, metabolic, and nonsurgical support of the bariatric surgery patient‐‐2013 update: cosponsored by American Association of Clinical Endocrinologists, The Obesity Society, and American Society for Metabolic & Bariatric Surgery. Obesity (Silver Spring). 2013;21(Suppl 1):S1‐S27. 23529939 10.1002/oby.20461 PMC4142593

[64] Kumari A , Nigam A . Bariatric surgery in women: a boon needs special care during pregnancy. J Clin Diagn Res. 2015;9(11):Qe01‐Qe05. 10.7860/JCDR/2015/14258.6802 PMC4668489 26672514

[65] Tack J , Arts J , Caenepeel P , De Wulf D , Bisschops R . Pathophysiology, diagnosis and management of postoperative dumping syndrome. Nat Rev Gastroenterol Hepatol. 2009;6(10):583‐590. 19724252 10.1038/nrgastro.2009.148

[66] van Beek AP , Emous M , Laville M , Tack J . Dumping syndrome after esophageal, gastric or bariatric surgery: pathophysiology, diagnosis, and management. Obes Rev. 2017;18(1):68‐85. 27749997 10.1111/obr.12467

[67] Department of Health and Social Care . Alcohol consumption: advice on low risk drinking. 2016; https://www.gov.uk/government/publications/alcohol-consumption-advice-on-low-risk-drinking.

[68] Sarwar H , Chapman WH 3rd , Pender JR , et al. Hypoglycemia after Roux‐en‐Y gastric bypass: the BOLD experience. Obes Surg. 2014;24(7):1120‐1124. 24737312 10.1007/s11695-014-1260-8

[69] Marsk R , Jonas E , Rasmussen F , Naslund E . Nationwide cohort study of post‐gastric bypass hypoglycaemia including 5,040 patients undergoing surgery for obesity in 1986‐2006 in Sweden. Diabetologia. 2010;53(11):2307‐2311. 20495972 10.1007/s00125-010-1798-5

[70] Kellogg TA , Bantle JP , Leslie DB , et al. Postgastric bypass hyperinsulinemic hypoglycemia syndrome: characterization and response to a modified diet. Surg Obes Relat Dis: official journal of the American Society for Bariatric Surgery. 2008;4(4):492‐499. 10.1016/j.soard.2008.05.005 18656831

[71] Eisenberg D , Azagury DE , Ghiassi S , Grover BT , Kim JJ . ASMBS position statement on postprandial hyperinsulinemic hypoglycemia after bariatric surgery. Surg Obes Relat Dis: official journal of the American Society for Bariatric Surgery. 2017;13(3):371‐378. 10.1016/j.soard.2016.12.005 28110984

[72] Salehi M , Vella A , McLaughlin T , Patti M‐E . Hypoglycemia after gastric bypass surgery: current concepts and controversies. J Clin Endocrinol Metabol. 2018;103(8):2815‐2826. 10.1210/jc.2018-00528 PMC6692713 30101281

[73] Devlieger R , Guelinckx I , Jans G , Voets W , Vanholsbeke C , Vansant G . Micronutrient levels and supplement intake in pregnancy after bariatric surgery: a prospective cohort study. PLoS ONE. 2014;9(12):e114192. 25470614 10.1371/journal.pone.0114192 PMC4254913

[74] Rothman KJ , Moore LL , Singer MR , Nguyen US , Mannino S , Milunsky A . Teratogenicity of high vitamin A intake. N Engl J Med. 1995;333(21):1369‐1373. 7477116 10.1056/NEJM199511233332101

[75] Food Standards Agency . Safer upper limits for vitamins and minerals. Expert group on vitamins and minerals. 2003.

[76] Cruz S , Matos A , da Cruz SP , Pereira S , Saboya C , Ramalho A . Relationship between the nutritional status of vitamin a per trimester of pregnancy with maternal anthropometry and anemia after Roux‐en‐Y gastric bypass. Nutrients. 2017;9(9):989. 10.3390/nu9090989 PMC5622749 28885564

[77] Jans G , Matthys C , Bogaerts A , et al. Maternal micronutrient deficiencies and related adverse neonatal outcomes after bariatric surgery: a systematic review. Adv Nutr. 2015;6(4):420‐429. 26178026 10.3945/an.114.008086 PMC4496736

[78] Faintuch J , Dias MC , de Souza FE , et al. Pregnancy nutritional indices and birth weight after Roux‐en‐Y gastric bypass. Obes Surg. 2009;19(5):583‐589. 18953618 10.1007/s11695-008-9755-9

[79] Bebber FE , Rizzolli J , Casagrande DS , et al. Pregnancy after bariatric surgery: 39 pregnancies follow‐up in a multidisciplinary team. Obes Surg. 2011;21(10):1546‐1551. 20820939 10.1007/s11695-010-0263-3

[80] Machado SN , Pereira S , Saboya C , Saunders C , Ramalho A . Influence of Roux‐en‐Y gastric bypass on the nutritional status of vitamin A in pregnant women: a comparative study. Obes Surg. 2016;26(1):26‐31. 25994779 10.1007/s11695-015-1734-3

[81] da Cruz SP , Matos A , Pereira S , Saboya C , da Cruz SP , Ramalho A . Roux‐en‐Y Gastric bypass aggravates vitamin A deficiency in the mother‐child group. Obes Surg. 2018;28(1):114‐121. 28676956 10.1007/s11695-017-2791-6

[82] Pietersma‐de Bruyn AL , van Haard PM , Beunis MH , Hamulyak K , Kuijpers JC . Vitamin K1 levels and coagulation factors in healthy term newborns till 4 weeks after birth. Haemostasis. 1990;20(1):8‐14. 2323682 10.1159/000216099

[83] Jans G , Guelinckx I , Voets W , et al. Vitamin K1 monitoring in pregnancies after bariatric surgery: a prospective cohort study. Surg Obes Relat Dis: official journal of the American Society for Bariatric Surgery. 2014;10(5):885‐890. 10.1016/j.soard.2014.04.032 25264330

[84] BOMSS . Guidelines on perioperative and postoperative biochemical monitoring and micronutrient replacement for patients undergoing bariatric surgery. 2014. 10.1111/obr.13087 PMC7583474 32743907

[85] Landais A . Neurological complications of bariatric surgery. Obes Surg. 2014;24(10):1800‐1807. 25060718 10.1007/s11695-014-1376-x

[86] Kuhn AL , Hertel F , Boulanger T , Diederich NJ . Vitamin B1 in the treatment of Wernicke's encephalopathy due to hyperemesis after gastroplasty. J Clin Neurosci. 2012;19(9):1303‐1305. 22727204 10.1016/j.jocn.2011.11.030

[87] Saab RO , El Khoury MI , Jabbour RA . Wernicke encephalopathy after Roux‐en‐Y gastric bypass and hyperemesis gravidarum. Surg Obes Relat Dis: official journal of the American Society for Bariatric Surgery. 2013;9(6):e105‐e107. 10.1016/j.soard.2013.05.002 23932007

[88] Stroh C , Meyer F , Manger T . Beriberi, a Severe Complication after Metabolic Surgery ‐ Review of the Literature. Obes Facts. 2014;7(4):246‐252. 25095897 10.1159/000366012 PMC5644786

[89] Jans G , Devlieger R , De Preter V , et al. Bariatric surgery does not appear to affect women's breast‐milk composition. J Nutr. 2018;148(7):1096‐1102. 29901782 10.1093/jn/nxy085

[90] Jans G , Matthys C , Lannoo M , Van der Schueren B , Devlieger R . Breast milk macronutrient composition after bariatric surgery. Obes Surg. 2015;25(5):938‐941. 25691352 10.1007/s11695-015-1610-1

[91] Gimenes JC , Nicoletti CF , de Souza Pinhel MA , Cortes‐Oliveira C , Salgado Junior W , Nonino CB . Nutritional status of children from women with previously bariatric surgery. Obes Surg. 2018;28(4):990‐995. 28980121 10.1007/s11695-017-2950-9

[92] WHO . Breastfeeding. 2018; https://www.who.int/topics/breastfeeding/en/, 2018.

[93] Martens WS 2nd , Martin LF , Berlin CM Jr . Failure of a nursing infant to thrive after the mother's gastric bypass for morbid obesity. Pediatrics. 1990;86(5):777‐778. 2235232

[94] Celiker MY , Chawla A . Congenital B12 deficiency following maternal gastric bypass. J Perinatol. 2009;29(9):640‐642. 19710657 10.1038/jp.2009.16

[95] Monnier D , Goulenok T , Allary J , Zarrouk V , Fantin B . Starvation ketosis in a breastfeeding woman. Rev Med Interne. 2015;36(12):854‐858. 25912080 10.1016/j.revmed.2015.03.011

[96] Galazis N , Docheva N , Simillis C , Nicolaides KH . Maternal and neonatal outcomes in women undergoing bariatric surgery: a systematic review and meta‐analysis. Eur J Obstet Gynecol Reprod Biol. 2014;181:45‐53. 25126981 10.1016/j.ejogrb.2014.07.015

[97] Yi XY , Li QF , Zhang J , Wang ZH . A meta‐analysis of maternal and fetal outcomes of pregnancy after bariatric surgery. Int J Gynaecol Obstet. 2015;130(1):3‐9. 25863541 10.1016/j.ijgo.2015.01.011

[98] Chevrot A , Kayem G , Coupaye M , Lesage N , Msika S , Mandelbrot L . Impact of bariatric surgery on fetal growth restriction: experience of a perinatal and bariatric surgery center. Am J Obstet Gynecol. 2016;214(5):655 e651‐655 e657. 26627725 10.1016/j.ajog.2015.11.017

[99] Cornthwaite K , Jefferys A , Lenguerrand E , et al. One size does not fit all. Management of the laparoscopic adjustable gastric band in pregnancy: a national prospective cohort study. The Lancet. 2015;385:S32. 10.1016/S0140-6736(15)60347-6 26312854

[100] Cools M , Duval EL , Jespers A . Adverse neonatal outcome after maternal biliopancreatic diversion operation: report of nine cases. Eur J Pediatr. 2006;165(3):199‐202. 16416132 10.1007/s00431-005-0056-1

[101] Smets KJ , Barlow T , Vanhaesebrouck P . Maternal vitamin A deficiency and neonatal microphthalmia: complications of biliopancreatic diversion? Eur J Pediatr. 2006;165(7):502‐504. 16718478 10.1007/s00431-006-0120-5

[102] Moliterno JA , DiLuna ML , Sood S , Roberts KE , Duncan CC . Gastric bypass: a risk factor for neural tube defects? Case report. J Neurosurg Pediatr. 2008;1(5):406‐409. 18447680 10.3171/PED/2008/1/5/406

[103] Van Mieghem T , Van Schoubroeck D , Depiere M , Debeer A , Hanssens M . Fetal cerebral hemorrhage caused by vitamin K deficiency after complicated bariatric surgery. Obstet Gynecol. 2008;112(2 Pt 2):434‐436. 18669754 10.1097/AOG.0b013e3181649e7b

[104] Kang L , Marty D , Pauli RM , Mendelsohn NJ , Prachand V , Waggoner D . Chondrodysplasia punctata associated with malabsorption from bariatric procedures. Surg Obes Relat Dis: official journal of the American Society for Bariatric Surgery. 2010;6(1):99‐101. 10.1016/j.soard.2009.05.004 19640801

[105] Sjöström L , Lindroos A‐K , Peltonen M , et al. Lifestyle, diabetes, and cardiovascular risk factors 10 years after bariatric surgery. N Engl J Med. 2004;351(26):2683‐2693. 15616203 10.1056/NEJMoa035622

[106] Benotti PN , Forse RA . The role of gastric surgery in the multidisciplinary management of severe obesity. Am J Surg. 1995;169(3):361‐367. 7879845 10.1016/s0002-9610(99)80177-9

[107] Christou NV , Look D , Maclean LD . Weight gain after short‐ and long‐limb gastric bypass in patients followed for longer than 10 years. Ann Surg. 2006;244(5):734‐740. 17060766 10.1097/01.sla.0000217592.04061.d5 PMC1856611

[108] Goldstein RF , Abell SK , Ranasinha S , et al. Association of gestational weight gain with maternal and infant outcomes: a systematic review and meta‐analysis. JAMA. 2017;317(21):2207‐2225. 28586887 10.1001/jama.2017.3635 PMC5815056

[109] Dixon JB , Dixon ME , O'Brien PE . Pregnancy after lap‐band surgery: management of the band to achieve healthy weight outcomes. Obes Surg. 2001;11(1):59‐65. 11361170 10.1381/096089201321454123

[110] Skull AJ , Slater GH , Duncombe JE , Fielding GA . Laparoscopic adjustable banding in pregnancy: safety, patient tolerance and effect on obesity‐related pregnancy outcomes. Obes Surg. 2004;14(2):230‐235. 15018752 10.1381/096089204322857618

[111] Ducarme G , Revaux A , Rodrigues A , Aissaoui F , Pharisien I , Uzan M . Obstetric outcome following laparoscopic adjustable gastric banding. Int J Gynaecol Obstet. 2007;98(3):244‐247. 17433814 10.1016/j.ijgo.2007.02.020

[112] Lapolla A , Marangon M , Dalfra MG , et al. Pregnancy outcome in morbidly obese women before and after laparoscopic gastric banding. Obes Surg. 2010;20(9):1251‐1257. 20524157 10.1007/s11695-010-0199-7

[113] Berglind D , Willmer M , Naslund E , Tynelius P , Sorensen TI , Rasmussen F . Differences in gestational weight gain between pregnancies before and after maternal bariatric surgery correlate with differences in birth weight but not with scores on the body mass index in early childhood. Pediatr Obes. 2014;9(6):427‐434. 24339139 10.1111/j.2047-6310.2013.00205.x

[114] Sheiner E , Balaban E , Dreiher J , Levi I , Levy A . Pregnancy outcome in patients following different types of bariatric surgeries. Obes Surg. 2009;19(9):1286‐1292. 19618246 10.1007/s11695-009-9920-9

[115] Santulli P , Mandelbrot L , Facchiano E , et al. Obstetrical and neonatal outcomes of pregnancies following gastric bypass surgery: a retrospective cohort study in a French referral centre. Obes Surg. 2010;20(11):1501‐1508. 20803358 10.1007/s11695-010-0260-6

[116] IOM (Institute of Medicine) and NRC (National Research Council) . Weight Gain During Pregnancy: Reexamining the Guidelines. Washington, DC: The National Academies Press; 2009. 20669500

[117] Jasaitis Y , Sergent F , Bridoux V , Paquet M , Marpeau L , Teniere P . Management of pregnancies after adjustable gastric banding. J Gynecol Obstet Biol Reprod (Paris). 2007;36(8):764‐769. 17512137 10.1016/j.jgyn.2007.03.010

[118] Burke AE , Bennett WL , Jamshidi RM , et al. Reduced Incidence of Gestational Diabetes with Bariatric Surgery. J Am Coll Surg. 2010;211(2):169‐175. 20670854 10.1016/j.jamcollsurg.2010.03.029

[119] Carreau AM , Nadeau M , Marceau S , Marceau P , Weisnagel SJ . Pregnancy after bariatric surgery: balancing risks and benefits. Can J Diabetes. 2017;41(4):432‐438. 28365201 10.1016/j.jcjd.2016.09.005

[120] Schaefer UM , Songster G , Xiang A , Berkowitz K , Buchanan TA , Kjos SL . Congenital malformations in offspring of women with hyperglycemia first detected during pregnancy. Am J Obstet Gynecol. 1997;177(5):1165‐1171. 9396914 10.1016/s0002-9378(97)70035-8

[121] Adam S , Ammori B , Soran H , Syed AA . Pregnancy after bariatric surgery: screening for gestational diabetes. BMJ. 2017;356:j533. 28159743 10.1136/bmj.j533

[122] Cosson E , Pigeyre M , Ritz P . Diagnosis and management of patients with significantly abnormal glycaemic profiles during pregnancy after bariatric surgery: PRESAGE (Pregnancy with significantly abnormal glycaemic exposure—bariatric patients). Diabetes Metab. 2017;44(4):376. 28988697 10.1016/j.diabet.2017.08.001

[123] American Diabetes A . Standards of medical care in diabetes—2013. Diabetes Care. 2013;36(Suppl 1(Suppl 1)):S11‐S66. 23264422 10.2337/dc13-S011 PMC3537269

[124] HAPO Study Cooperative Research Group . Hyperglycemia and adverse pregnancy outcomes. N Engl J Med. 2008;358(19):1991‐2002. 18463375 10.1056/NEJMoa0707943

[125] Gobl CS , Bozkurt L , Tura A , et al. Assessment of glucose regulation in pregnancy after gastric bypass surgery. Diabetologia. 2017;60(12):2504‐2513. 28918470 10.1007/s00125-017-4437-6 PMC6448941

[126] National Institute for Health and Care Excellence . Diabetes in pregnancy: management from preconception to the postnatal period. NICE guideline [NG3]. 2015; https://www.nice.org.uk/guidance/ng3. 31846258

[127] Standards of Medical Care in Diabetes—2017: Summary of Revisions. Diabetes Care. 2017;40(Supplement 1):S4‐S5. 27979887 10.2337/dc17-S003

[128] Bhatti JA , Nathens AB , Thiruchelvam D , Grantcharov T , Goldstein BI , Redelmeier DA . Self‐harm emergencies after bariatric surgery. JAMA Surg. 2016;151(3):226. 26444444 10.1001/jamasurg.2015.3414

[129] Adams TD , Gress RE , Smith SC , et al. Long‐term mortality after gastric bypass surgery. N Engl J Med. 2007;357(8):753‐761. 17715409 10.1056/NEJMoa066603

[130] Östlund MP , Backman O , Marsk R , et al. Increased admission for alcohol dependence after gastric bypass surgery compared with restrictive bariatric surgery. JAMA Surg. 2013;148(4):374. 23716012 10.1001/jamasurg.2013.700

[131] Jans G , Matthys C , Bogaerts A , et al. Depression and anxiety: lack of associations with an inadequate diet in a sample of pregnant women with a history of bariatric surgery—a Multicenter Prospective Controlled Cohort Study. Obes Surg. 2018;28(6):1629‐1635. 29230623 10.1007/s11695-017-3060-4

[132] Devlieger RJG , Matthys C . Outcomes of pregnancy after bariatric surgery. N Engl J Med. 2015;372(23):2266‐2268. 10.1056/NEJMc1503863 26039607

[133] Petersen L , Lauenborg J , Svare J , Nilas L . The impact of upper abdominal pain during pregnancy following a gastric bypass. Obes Surg. 2017;27(3):688‐693. 27568032 10.1007/s11695-016-2339-1

[134] Leal‐Gonzalez R , De La Garza‐Ramos R , Guajardo‐Perez H , Ayala‐Aguilera F , Rumbaut R . Internal hernias in pregnant women with history of gastric bypass surgery: case series and review of literature. Int J Surg Case Rep. 2013;4(1):44‐47. 23108170 10.1016/j.ijscr.2012.10.006 PMC3537949

[135] Vannevel V , Jans G , Bialecka M , Lannoo M , Devlieger R , Van Mieghem T . Internal herniation in pregnancy after gastric bypass: a systematic review. Obstet Gynecol. 2016;127(6):1013‐1020. 27159745 10.1097/AOG.0000000000001429

[136] Wax JR , Pinette MG , Cartin A . Roux‐en‐Y gastric bypass‐associated bowel obstruction complicating pregnancy—an obstetrician's map to the clinical minefield. Am J Obstet Gynecol. 2013;208(4):265‐271. 22964065 10.1016/j.ajog.2012.08.014

[137] Carelli AM , Ren CJ , Youn HA , et al. Impact of laparoscopic adjustable gastric banding on pregnancy, maternal weight, and neonatal health. Obes Surg. 2011;21(10):1552‐1558. 20835780 10.1007/s11695-010-0265-1

[138] Haward RN , Brown WA , O'Brien PE . Does pregnancy increase the need for revisional surgery after laparoscopic adjustable gastric banding? Obes Surg. 2011;21(9):1362‐1369. 20680505 10.1007/s11695-010-0235-7

